# Whole blood gene expression in adolescent chronic fatigue syndrome: an exploratory cross-sectional study suggesting altered B cell differentiation and survival

**DOI:** 10.1186/s12967-017-1201-0

**Published:** 2017-05-11

**Authors:** Chinh Bkrong Nguyen, Lene Alsøe, Jessica M. Lindvall, Dag Sulheim, Even Fagermoen, Anette Winger, Mari Kaarbø, Hilde Nilsen, Vegard Bruun Wyller

**Affiliations:** 1 Department of Paediatrics and Adolescent Health, Akershus University Hospital, 1478 Lørenskog, Norway; 2 Division of Medicine and Laboratory Sciences, Medical Faculty, University of Oslo, Oslo, Norway; 3 Institute of Clinical Medicine, Department of Clinical Molecular Biology, University of Oslo, and Akershus University Hospital, Lørenskog, Norway; 4 National Bioinformatics Infrastructure Sweden (NBIS), Science for Life Laboratory, Department of Biochemistry and Biophysics, Stockholm University, Stockholm, Sweden; 5 Department of Paediatrics, Lillehammer County Hospital, Lillehammer, Norway; 6 Department of Anesthesiology and Critical Care, Oslo University Hospital, Oslo, Norway; 7 Institute of Nursing Sciences, Oslo and Akershus University College of Applied Sciences, Oslo, Norway; 8 Department of Microbiology, Oslo University Hospital, Oslo, Norway

**Keywords:** Chronic fatigue syndrome, Adolescent, Gene expression, Inflammation, B cell differentiation, B cell survival

## Abstract

**Background:**

Chronic fatigue syndrome (CFS) is a prevalent and disabling condition affecting adolescents. The pathophysiology is poorly understood, but immune alterations might be an important component. This study compared whole blood gene expression in adolescent CFS patients and healthy controls, and explored associations between gene expression and neuroendocrine markers, immune markers and clinical markers within the CFS group.

**Methods:**

CFS patients (12–18 years old) were recruited nation-wide to a single referral center as part of the NorCAPITAL project. A broad case definition of CFS was applied, requiring 3 months of unexplained, disabling chronic/relapsing fatigue of new onset, whereas no accompanying symptoms were necessary. Healthy controls having comparable distribution of gender and age were recruited from local schools. Whole blood samples were subjected to RNA sequencing. Immune markers were blood leukocyte counts, plasma cytokines, serum C-reactive protein and immunoglobulins. Neuroendocrine markers encompassed plasma and urine levels of catecholamines and cortisol, as well as heart rate variability indices. Clinical markers consisted of questionnaire scores for symptoms of post-exertional malaise, inflammation, fatigue, depression and trait anxiety, as well as activity recordings.

**Results:**

A total of 29 CFS patients and 18 healthy controls were included. We identified 176 genes as differentially expressed in patients compared to controls, adjusting for age and gender factors. Gene set enrichment analyses suggested impairment of B cell differentiation and survival, as well as enhancement of innate antiviral responses and inflammation in the CFS group. A pattern of co-expression could be identified, and this pattern, as well as single gene transcripts, was significantly associated with indices of autonomic nervous activity, plasma cortisol, and blood monocyte and eosinophil counts. Also, an association with symptoms of post-exertional malaise was demonstrated.

**Conclusion:**

Adolescent CFS is characterized by differential gene expression pattern in whole blood suggestive of impaired B cell differentiation and survival, and enhanced innate antiviral responses and inflammation. This expression pattern is associated with neuroendocrine markers of altered HPA axis and autonomic nervous activity, and with symptoms of post-exertional malaise.

*Trial registration* Clinical Trials NCT01040429

**Electronic supplementary material:**

The online version of this article (doi:10.1186/s12967-017-1201-0) contains supplementary material, which is available to authorized users.

## Background

Chronic fatigue syndrome (CFS) is a long-lasting and disabling condition characterized by disproportional fatigue after exertions, musculoskeletal pain, headaches, cognitive impairments, and other symptoms [1, 2]. Adolescent CFS prevalence is estimated at 0.1–1.0% [3–5], and CFS may have detrimental effects on psychosocial and academic development [6], as well as family functioning [7].

The disease mechanisms of CFS remain poorly understood, but some studies indicate modest immunological alterations, such as low-grade systemic inflammation and attenuation of NK cell function [8–10]. Furthermore, the reported beneficial effect of treatment with the anti-CD20 antibody *rituximab* might suggest a role for B cells in the pathophysiology [11]. Studies of plasma cytokine levels have been inconclusive; findings include increased levels of interleukin (IL)-1 and tumor necrosis factor (TNF) [12], increased levels of IL-1α and IL-1β but normal levels of TNF [13], and no differences between CFS patients and healthy controls [14, 15].

Immune cell gene expression has been addressed by several studies over the last decade. However, the findings do not give a consistent picture: Kerr and co-workers reported differential expression of 88 genes in whole blood samples from CFS patients and healthy controls [16]. A similar pattern of gene expression was later found in two other CFS patient cohorts by the same research group [17]. From leukocyte samples, Light and co-workers reported an increase in expression of genes that are related to sensory, adrenergic and immune system as a response to physical exercise in CFS patients but not in healthy controls [18]. A recent review concluded that there is a larger post-exercise increase in *IL*-*10* and Toll-like receptor 4 (*TLR4*) gene transcripts in CFS as compared to healthy controls [19]. Restricting the analyses to gene expression from peripheral blood mononuclear cells (PBMC) correlated with multidimensional fatigue inventory and depression scales, Fang and co-workers identified cytokine–cytokine receptor interaction as one of the most significant pathways [20]. Also studying PBMC, Gow and co-workers identified that the top upregulated genes are related to immunological processes [21]. On the other hand, a study of monozygotic twins discordant for CFS did not reveal any differences in whole blood gene expression [22], and it has been maintained that previously reported differences in gene expression were study-specific and not useful for CFS diagnostic purposes [23]. Also, attempts of relating gene expression profiles to clinical symptoms of CFS have had limited success [24]. For instance, Galbraith and co-workers investigated whole blood gene expression in three post-infective cohorts; 63 genes were identified as differentially expressed, but there were no consistent associations to clinical symptoms [25].

The reasons for these discrepancies may partly be due to the multifactorial nature of CFS, which may obscure direct correlations with molecular observations. The complex regulation of transcription, post transcriptional control and RNA metabolism may also prompt variability in gene expression studies; hence mRNA measurements are not always linearly correlated with targeted functional proteins in biological samples at varying time-points.

In addition to immune changes, some studies have found that CFS disease mechanisms are characterized by neuroendocrine alterations including enhanced sympathetic and attenuated parasympathetic cardiovascular nervous activity [26–29] and attenuation of the hypothalamus–pituitary–adrenal axis (HPA axis) [30–32]. These phenomena might be causally related. The complex immune influence exerted by glucocorticoids has been recognized for decades [33]; more recently, ample evidence suggests that both parasympathetic and sympathetic nervous activity promotes immunomodulation [34–36]. Accordingly, the “sustained arousal” model of CFS suggests that the observed immune alterations are secondary to the neuroendocrine alterations [37]. This hypothesis received some support from the observation that treatment of adolescent CFS patients with low-dose *clonidine*, which attenuates sympathetic and enhanced parasympathetic nervous activity through central mechanisms [38], caused a significant reduction in serum levels of C-reactive protein (CRP) [39].

To the best of our knowledge, no previous study has addressed whole blood gene expression in adolescent CFS patients, who are less burdened by comorbidity and aging processes and presumably more homogeneous than adult patients. Nor do we know of any study using high throughput sequencing (HTS) for gene expression analyses in CFS. Furthermore, no previous study has explored associations between neuroendocrine markers and gene expression in CFS. Thus, the aim of this exploratory study was twofold: (a) To map whole blood differential gene expression in adolescent CFS patients and healthy controls, and (b) To explore the associations between gene expression and neuroendocrine markers, immune markers and clinical markers within the CFS group.

## Methods

### CFS patients

This study is part of the NorCAPITAL-project (The Norwegian Study of Chronic Fatigue Syndrome in Adolescents: Pathophysiology and Intervention Trial; ClinicalTrials ID: NCT01040429). Details of the recruitment procedure and inclusion/exclusion criteria are described elsewhere [39]. Briefly, all hospital paediatric departments in Norway (n = 20), as well as primary care paediatricians and general practitioners, were invited to refer CFS patients aged 12–18 years consecutively to our study center. A standard form required the referral unit to confirm the result of clinical investigations considered compulsory to diagnose pediatric CFS according to national Norwegian recommendations. Exclusion criteria encompassed somatic and psychiatric co-morbidity, pharmaceutical usage (including hormone contraceptives) and being bed-ridden. Patients considered eligible to this study were summoned to a clinical encounter at our study center after which a final decision on inclusion was made.

In agreement with clinical guidelines [2, 40] and previous studies from our group [27–29], we applied a ‘broad’ case definition of CFS, requiring 3 months of unexplained, disabling chronic/relapsing fatigue of new onset. We did not require that patients meet any other accompanying symptom criteria.

### Healthy controls

A group of healthy controls with a comparable distribution of gender and age were recruited from local schools. Controls were not matched to cases on any variable. No chronic disease and no regular use of pharmaceuticals (including hormone contraceptives) were allowed.

### Study design and ethics

A 1-day in-hospital assessment included clinical examination and blood sampling and always commenced between 7.30 and 9.30 a.m. All participants were instructed to fast overnight and abstain from tobacco products and caffeine for at least 48 h. The participants were instructed to apply an ointment containing the local anesthetic lidocaine (Emla^®^) on the skin in the antecubital area 1 h in advance. After at least 5 min supine rest in calm surroundings, blood samples were obtained in a fixed sequence from antecubital venous puncture. A questionnaire was completed after the clinical encounter and returned in a pre-stamped envelope.

Data were collected in the period from March 2010 until October 2012. The NorCAPITAL project has been approved by the Norwegian National Committee for Ethics in Medical Research and the Norwegian Medicines Agency. Written informed consent was obtained from all participants and from parents/next-of-kin if required. Details of the design are reported elsewhere [39].

### Gene expression profiling by RNA sequencing

Whole blood samples (3 mL) at baseline were collected and stored according to the protocol of the Invitrogen Tempus stabilizing reagents (Applied Biosystems, Thermo Fischer Scientific, Waltham, MA, USA). Total RNA was extracted using the Tempus Isolation kit according to manufacturer’s manual with the exception that 2 mL out of the 9 mL mixture of whole blood and reagent were extracted using a modified protocol where 3 mL blood was mixed well with 6 mL Invitrogen Tempus reagent and 2 mL of the mixture was used for RNA isolation. Removal of globin RNA was performed using the Human GLOBINclear kit (Ambion Inc., Texas, USA). The RNA sample quality was analyzed using the Lab-on-a-Chip Agilent RNA Nano kit (Agilent, Santa Clara, USA) and the Agilent 2100 Bioanalyzer platform. RNA samples with RNA integrity number (RIN) value ≥7 were used for gene expression characterization by RNA sequencing (RNA-Seq) at the Genomics Core Facilities at the Oslo University Hospital Radiumhospitalet, Norway. RNA library preparation and sequencing were performed according to the HiSeq 2500 Illumina protocol for 101 bp single-end strand-specific sequencing (Illumina Inc., San Diego, CA, USA). 130 ng of Globin depleted RNA from each sample was converted into a cDNA library using the RiboZero Gold and TruSeq Stranded mRNA Sample Prep Kit (Illumina Inc., San Diego, CA, USA). A total of 15–35 million reads were generated per sample.

### Transcriptome alignment and gene expression quantification

Raw RNA reads from Illumina sequencing were assessed by the fastQC tool [41] to assess sequence quality per base, quality scores per sequence, sequence and GC content per base, sequence length distribution, sequence duplication levels, Kmer content and overrepresented sequences (which also detected the presentation of ribosomal contamination). Adapter contamination elimination and reads trimming were conducted by the fastx toolkit [42].

All reads that passed QC assessment were mapped to the human genome version GRCh38.p2 by STAR [43]. To investigate the level and uniformity of the read coverage against the human genome, we plotted mapped reads against all human chromosomes using the SeqMonk software [41].

Statistics for differential expression analyses were performed using Bioconductor tools [44] in the R environment version 3.1.2. Gene expression abundance was quantified by the Subread package [45] at the gene level. Normalization of raw read quantification and removal of variation before differential expression analyses were processed following RUVg method [46]. Differentially expressed genes (DEG) between CFS patients and controls were identified using DESeq2 package [47]. In order to correct for possible confounding background factors, age groups as scaling factor and gender input were included in the design model of DESeq2. For each DEG, a p value cut off ≤0.10 after multiple-testing adjustment by Benjamini–Hochberg [False Discovery Rate (FDR) 10%] was applied, in accordance with the DESeq2 workflow.

A heatmap of samples distance was constructed by clustering distance matrix from logarithm 2 transformed values of count data [48] using the *pheatmap* package of Bioconductor. Hierarchical clustering of 100 top DEGs was performed using *genefilter* and *pheatmap* packages of Bioconductor in order to measure the deviation of expression value of each sample from the average expression across all samples. The purpose is to build blocks of genes that co-vary across different samples, and clustering the amount by which each gene deviates in a specific sample from the gene’s average across all samples.

### Validation of differentially expressed genes

To validate some of the genes from the DEG list, RT-qPCR was performed on the RNA material subjected to sequencing. Specific primers for each target gene were designed as to establish RT-qPCR conditions for each DEG individually (Additional file 1: Table S1). RNA was converted into cDNA by High-Capacity cDNA Reverse Transcription Kit (Life Technologies, Carlsbad, CA, US). Five nanogram cDNA was tested in duplicate reaction on a 7900 HT real-time machine (Applied Biosystems, Foster City, California, USA), using the Evagreen Sso Fast Master mix (Biorad Laboratories, CA, USA). The relative expression levels of each DEG were calculated by the 2^ΔΔCt^ method and were normalized to the *GAPDH* reference gene.

### Downstream data analysis

Functional annotation of genes obtained from DESeq 2 was done by uploading all DEGs into HumanMine [49]. Network visualization and Functional Enrichment Analysis was conducted through Cytoscape software 3.3. and ClueGO 2.3.2 [50]. Log2 of fold change of the expression value (after normalization) was imported into QIAGEN Ingenuity Pathways Analysis (IPA) for an Upstream Transcriptional Factor analysis as well as a mechanistic network enrichment analysis.

Previous analyses of whole blood gene expression in CFS patients [51] as well as healthy individuals [52] have revealed that co-expression of genes is a common phenomenon. Such co-expression might be the effect of neuroendocrine signaling initiating a specific expression pattern; this is in line with the “sustained arousal”-model of CFS [37]. Furthermore, a certain pattern of co-expression might be associated with specific clinical phenomena. To explore different axis of co-expression and reduce dimensionality in the present study, a factor analyses [principal component analysis (PCA) featuring varimax rotation] was applied to the DEG dataset (RNA-Seq normalized counts), in line with previous reports [51, 52]. Thereafter, the associations between factor scores and immune, neuroendocrine and clinical markers (cf. below) were explored using correlation and regression analyses. Similar association studies were also performed for some selected single gene transcriptional counts. In all these analyses, a p ≤ 0.05 was considered statistically significant; no adjustment for multiple testing was performed.

### Immune markers

Serum samples from 21 CFS patients and 18 controls were used to identify levels of immunoglobulins. The immunoglobulin classes IgA, IgE, IgM and the four IgG subclasses IgG_1_, IgG_2_, IgG_3_ and IgG_4_ in serum were measured using Luminex bead-based multiplex technology with reagents from the Procartaplex Immunoassay (Affymetrix eBioscience, San Diego, USA). The concentration of each sample was determined by plotting the expected concentration of standards against fluorescence intensity. Data analysis was performed using Procartaplex Analyst 1.0 and normalization was based on the best curve fit of standards curve.

The serum concentration of C-reactive protein (CRP) was analyzed as described previously [39]. Blood samples for analyses of IL-1β, IL-6 and TNF were placed on ice; plasma was separated by centrifugation (2500×*g*, 10 min, 4 °C) and frozen at −80 °C until assayed using a multiplex cytokine assay (Bio-Plex Human Cytokine 27-Plex Panel; Bio-Rad Laboratories Inc., Hercules, CA, USA) as described elsewhere [15]. Hematology and biochemistry routine assays were performed at the accredited laboratory at Oslo University Hospital, Norway.

### Neuroendocrine markers

As outlined in detail elsewhere [32], blood samples for plasma norepinephrine (NE) and epinephrine (E) were placed on ice; thereafter, plasma was separated by centrifugation (2250×*g*, 15 min, 4 °C) and assayed by high-performance liquid chromatography (HPLC) with a reversed-phase column and glassy carbon electrochemical detector (Antec, Leyden Deacade II SCC, Zoeterwoude, The Netherlands) using a commercial kit (Chromsystems, München, Germany). Plasma cortisol level was determined by routine assays at the accredited laboratory at Oslo University Hospital, Norway. Morning spot urine samples for NE and E analyses were acidified to pH 2.5 immediately after collection, and assayed with the same HPLC protocol as for plasma measurements [32]. Morning spot urine free cortisol (non-conjugated cortisol) was assayed by solid phase competitive luminescence immunoassay (LIA) (type Immulite^®^ 2000, Siemens Healthcare Diagnostics, NY, USA). The urine levels of creatinine were analyzed using standard automatic analyzer techniques at the accredited laboratory at Oslo University Hospital, Norway.

Indices of heart rate variability (HRV) were obtained from ECG recordings of participants laying in a horizontal position and connected to the Task Force Monitor (TFM) (Model 3040i, CNSystems Medizintechnik, Graz, Austria). Methodological details are provided elsewhere [53]. Power spectral analysis of HRV was automatically provided by the TFM, returning numerical values for Low Frequency (LF) power (0.05–0.17 Hz), High Frequency (HF) power (0.17–0.4 Hz) and the LF/HF ratio. In addition, the time-domain index RMSSD (the square root of the mean square differences of successive RR-intervals) was computed. RMSSD and HF power are both considered indicative of parasympathetic heart rate modulation; LF power reflects the combined effect of sympathetic and parasympathetic heart rate control, whereas the LF/HF ratio is an index of sympathetic/parasympathetic balance [54].

### Clinical markers

A CFS symptom inventory for adolescents assesses the frequency of 24 common symptoms during the preceding month, as has been described elsewhere [39]. Briefly, each symptom is rated on a 5-point Likert scale, ranging from ‘never/rarely present’ to ‘present all the time’. A composite score reflecting inflammatory symptoms was generated by taking the arithmetic mean across three single items (fever/chills, sore throat, and tender lymphatic nodes) and a composite score reflecting symptoms of post-exertional malaise was generated by taking the arithmetic mean across two single items (post-exertional fatigue and non-refreshing sleep). For both variables, the total range is from 0 to 5; higher scores imply more severe symptom burden.

The Chalder Fatigue Questionnaire (CFQ) total sum score is applied in the present study [55]; total range is from 0 to 33, where higher scores imply more severe fatigue. The Mood and Feelings Questionnaire (MFQ) consists of 34 items, each scored on a 0–2 Likert scale; thus, the total sum score is from 0 to 68 [56]. The Spielberger State-Trait Anxiety Inventory subscore reflecting trait anxiety is derived from the sum across 20 items; total range is from 20 to 80 [57]. The *activPAL* accelerometer device (PAL Technologies Ltd, Glasgow, Scotland) was used for monitoring of daily physical activity during 7 consecutive days [58], as described elsewhere [39].

## Results

### Participants

RNA was extracted from a sub-cohort of the NorCAPITAL study and a total of 60 samples with RIN value ≥7 were subjected to RNA sequencing. After removing ribosomal contamination and bad quality reads from the RNA-Seq experiment, a random sample of 29 CFS patients and 18 healthy controls (a total of 47, mean RIN value = 7.67) were analyzed further for differential gene expression quantification in the present study.

The background characteristics of the two groups are given in Table 1. In line with previously reported findings from the NorCAPITAL project [39], plasma norepinephrine, plasma epinephrine, and urine norepinephrine were significantly higher in the CFS group, as were scores of symptoms of post-exertional malaise, inflammation, fatigue, depression, and trait anxiety. The number of steps per day was significantly lower in the CFS group. Overall, the values of the different variables in the present study are comparable to the values pertaining to the entire NorCAPITAL cohort (Additional file 2: Table S2), except for urine cortisol/creatinine ratio (for which there was no across-group difference in the present study but lower among CFS patients in the entire NorCAPITAL cohort).

**Table 1:** Background characteristics of the chronic fatigue syndrome (CFS) group and the healthy control (HC) group in the present study

	CFS group (n = 29)		HC group (n = 18)		p value^e^
Background markers					
Female gender. Number, %	18	62	11	61	0.948
Scandinavian ethnicity. Number, %	29	100	17	95	0.383
Age (years). Mean, SD	15.1	1.4	14.7	1.4	0.335
Body mass index (kg/m^2^). Mean, SD	20.2	3.4	19.4	1.9	0.317
Disease duration (months). Median, range	12	4–60	n/a		
Adheres to the Fukuda criteria of CFS^a^. Number, %	20	69	n/a		
Adheres to the Canada 2003-criteria of CFS^b^. Number, %	11	38	n/a		
Immune markers					
Blood leukocytes (cells × 10^9^/L). Mean, SD	6.0	2.0	5.5	1.0	0.370
Blood neutrophils (cells × 10^9^/L). Mean, SD	3.1	1.6	2.8	0.7	0.462
Blood lymphocytes (cells × 10^9^/L). Mean, SD	2.2	0.7	2.1	0.5	0.626
Blood monocytes (cells × 10^9^/L). Mean, SD	0.48	0.19	0.42	0.10	0.146
Blood eosinophils (cells × 10^9^/L). Mean, SD	0.18	0.11	0.17	0.07	0.787
Blood basophils (cells × 10^9^/L). Mean, SD	0.02	0.04	0.02	0.04	0.681
Serum C-reactive protein (mg/L). Median, IQR	0.40	0.89	0.32	0.28	0.405
Plasma interleukin-1β (pg/mL). Mean, SD	3.0	2.1	2.3	1.5	0.223
Plasma interleukin-6 (pg/mL). Mean, SD	10.0	7.5	7.2	4.3	0.158
Plasma tumor necrosis factor (pg/mL). Mean, SD	63	40	47	29	0.161
Neuroendocrine markers					
Plasma norepinephrine (pmol/L). Mean, SD	2067	835	1530	358	*0.004*
Plasma epinephrine (pmol/L). Mean, SD	362	131	284	74	*0.012*
Plasma cortisol (nmol/L). Mean, SD	334	151	349	202	0.782
Urine norepinephrine/creatinine ratio (nmol/mmol). Mean, SD	14.5	6.5	10.9	3.6	*0.033*
Urine epinephrine/creatinine ratio (nmol/mmol). Mean, SD	1.7	1.1	1.6	0.9	0.657
Urine cortisol/creatinine ratio (nmol/mmol). Median, IQR	4.4	3.3	4.5	2.8	0.605
Heart rate variability, RMSSD (ms). Mean, SD^c^	83	50	n/a		
Heart rate variability, LF power (abs). Median, IQR^d^	541	1068	844	1729	0.445
Heart rate variability, HF power (abs). Median, IQR^d^	919	2557	1009	1414	0.666
Heart rate variability, LF/HF-ratio. Mean, SD^d^	0.83	0.59	0.90	0.41	0.774
Clinical markers					
Inflammatory symptoms (total score). Mean, SD^d^	2.1	0.8	1.3	0.5	*0.010*
Symptoms of post-exertional malaise (total score). Median, IQR^d^	4.0	1.5	1.0	0.4	*<0.001*
Chalder fatigue questionnaire (total score). Mean, SD^d^	20.4	5.2	6.8	4.9	*<0.001*
Moods and feelings questionnaire (total score). Mean, SD^d^	20.6	10.8	3.9	3.8	*<0.001*
Spielberger state-trait anxiety questionnaire (trait subscore). Mean, SD^d^	46	9.1	32	3.2	*<0.001*
Steps per day (number). Mean, SD^d^	4698	2622	11,282	4670	*0.005*

Italics indicate a statistically significant p-value

*n/a* not applicable, *SD* standard deviation, *IQR* interquartile range, *RMSSD* square root of the mean squared differences of subsequent RR-intervals in the ECG, *LF* low-frequency power of heart rate variability, *HF* high-frequency power of heart rate variability

^a^Cf. Ref. [88]

^b^Cf. Ref. [89]

^c^In the present study, no data were obtained from the healthy control group

^d^In the present study, data were obtained from eight healthy controls only

^e^Based upon t test, Mann–Whitney test or Fisher exact test as appropriate

### Differentially expressed genes in whole blood between CFS patients and healthy controls

**Fig. 1:**
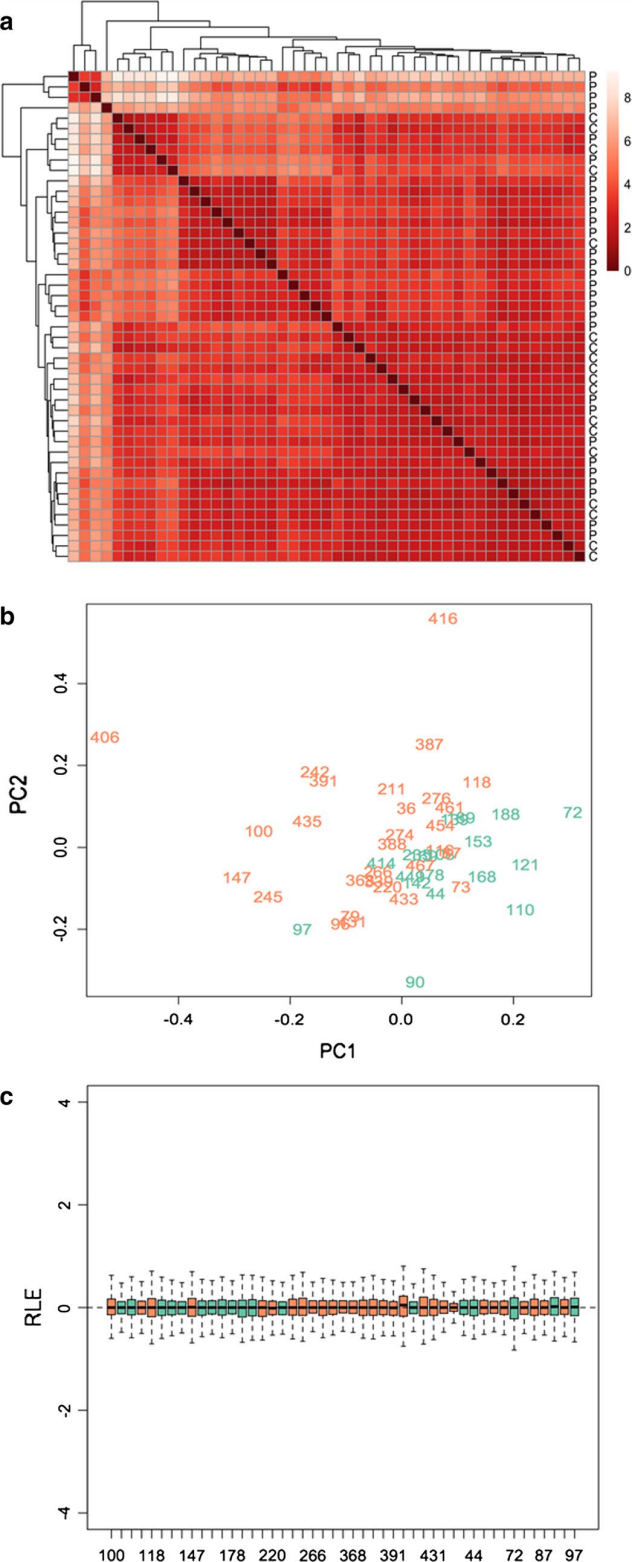
**a** Hierarchal clustering of all 47 samples based on the rlog value [48]. The color density at the *top right panel* reflects the Euclidean distance. *P* CFS patients, *C* healthy controls. **b** Output of variation removal of our RNA-Seq data using RUVSeq. principle component analysis (PCA) is performed without using any differently expressed genes, and demonstrates relatively good separation between CFS patients (*orange*) and healthy controls (*green*). **c** Relative log expression (RLE) plot shows the distribution of read counts across all samples centered around zero. The *y axis* corresponds to the deviation of each RLE per gene per sample compared to median RLE over all samples (*x axis*). (CFS patients *orange*. Healthy controls *green*)

RNA-Seq produced 18–45 × 10^6^ single end reads per sample, which was previously reported to be sufficient for transcriptome quantification [59]. The rate of unique mapping into the reference genome was 80–92%, with 50% reads mapped to exons. A percentage of the reads (3–5%) were found to be mapped to ribosomal RNAs. Multiply mapped reads, reads mapped to the sense strands and reads mapped to exon–exon boundaries were not counted. As might be expected from the inherent heterogeneity of whole-blood gene expression, there was no evident subgrouping between either patients or controls in our gene expression data before normalization. This is illustrated in Fig. 1a where the individual samples are distant from one another.

Normalization and differential expression analysis, with correction for age and gender factors, detected a total of 176 genes that were differentially expressed between CFS patients and healthy controls (adjusted p < 0.10) (Additional file 3: Table S3). The robustness of DEGs after normalization was confirmed by good separation between the CFS and control groups through principal component analysis (Fig. 1b) and by plotting regular log expression values compared with median of log expression across all samples (Fig. 1c).

**Fig. 2:**
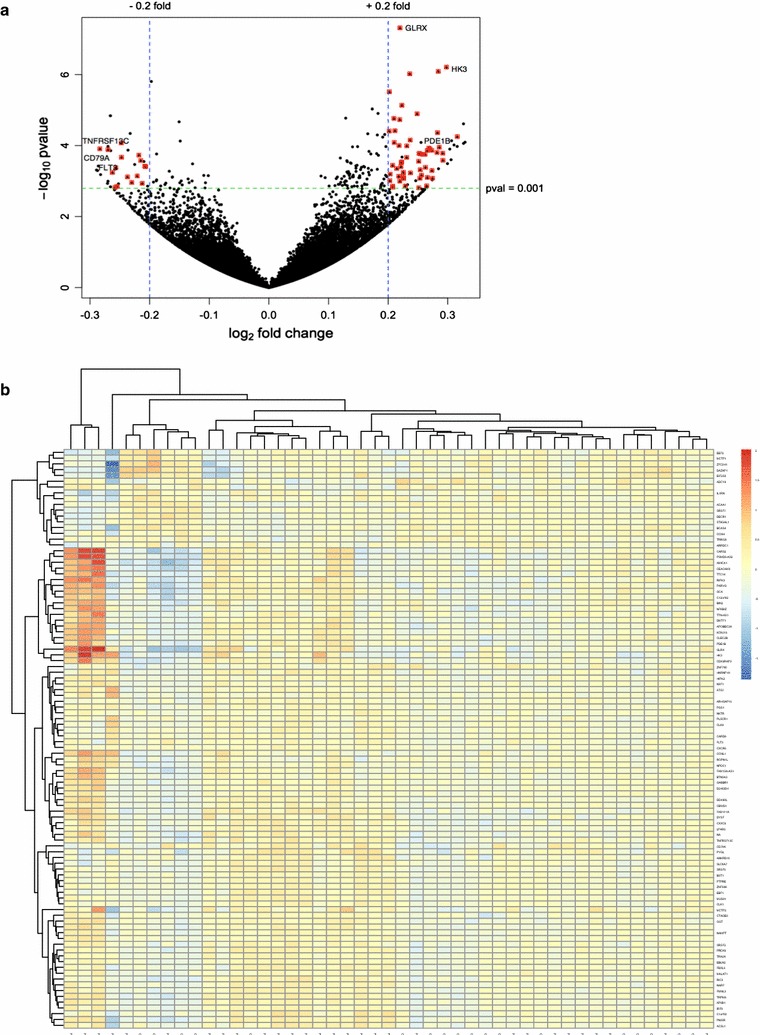
**a** Volcano plot showing the alignment between DESeq p values versus log2 fold changes of CFS patients against healthy controls. *Red points* indicate DEGs with a log2 fold change >0.2 and p < 0.0016 (Table 2). **b** Hierarchical clustering of all 176 differently expressed genes. The heatmap was constructed based on the deviation of gene expression levels of individual sample from averaged gene expression across all samples (Table 2). The color code for variance value is shown in the *upper right corner* of the panel

Of the 176 DEGs, 137 were upregulated and 37 were downregulated (Fig. 2a, b; Additional file 3: Table S3). This corresponds to an observation of 78% of the DEGs being up-regulated in CFS patients as compared to 22% of the genes having a down-regulated transcriptional pattern compared to healthy controls. Although significant, the differences in normalized expression levels were small, ranging from 0.8- to 1.25-fold (linear scale) (Table 2; Additional file 3: Table S3). Among the 176 differentially expressed genes we observed nuances of expression both within the groups as well as between the two groups (Fig. 2b).

**Table 2:** Differentially expressed immune genes, their annotated proteins, and their annotated biological processes based on gene ontologies in CFS patients as compared to healthy controls, adjusted for age and gender differences across groups and sorted according to foldchange

Differential expression	Gene name	Ensembl ID	Fold change	p value, unadjusted	p value, adjusted	Protein	Gene ontology biological process	Gene ontology identifier
Downregulated gene expression in CFS patients as compared to healthy controls	CD79A	ENSG00000105369	0.821	0.00012	0.0393	CD79a molecule	B cell differentiation	GO:0030183
							B cell proliferation	GO:0042100
							B cell activation	GO:0042113
							B cell receptor signaling pathway	GO:0050853
	TNFRSF13C	ENSG00000159958	0.829	0.00012	0.0395	Tumor necrosis factor receptor superfamily member 13C	B cell homeostasis	GO:0001782
	FLT3	ENSG00000122025	0.833	0.00055	0.0682	Fms related tyrosine kinase 3	Myeloid progenitor cell differentiation	GO:0002318
							Pro-B cell differentiation	GO:0002328
							Transmembrane receptor protein tyrosine kinase signaling pathway	GO:0007169
							Positive regulation of cell proliferation	GO:0008284
							Cytokine-mediated signaling pathway	GO:0019221
							B cell differentiation	GO:0030183
							Lymphocyte proliferation	GO:0046651
							Cellular response to cytokine stimulus	GO:0071345
							Cellular response to glucocorticoid stimulus	GO:0071385
	EBF1	ENSG00000164330	0.836	0.00041	0.0615	Early B cell factor 1	Multicellular organism development	GO:0032501
	CXCR5	ENSG00000160683	0.848	0.00073	0.0735	C-X-C motif chemokine receptor 5	Positive regulation of cytokinesis	GO:0032467
							B cell activation	GO:0042113
							Lymph node development	GO:0048535
							Chemokine-mediated signaling pathway	GO:0070098
	IRF4	ENSG00000137265	0.863	0.00116	0.0879	Interferon regulatory factor 4	Negative regulation of toll-like receptor signaling pathway	GO:0034122
	HIPK2	ENSG00000064393	0.891	0.00054	0.0682	Homeodomain interacting protein kinase 2	Positive regulation of cell proliferation	GO:0008284
							Positive regulation of cytokine production	GO:0001819
							Activation of innate immune response	GO:0002218
							Inflammatory response	GO:0006954
							Myeloid cell differentiation	GO:0030099
							Monocyte differentiation	GO:0030224
							Positive regulation of type I interferon production	GO:0032481
							Positive regulation of interleukin-1 beta production	GO:0032731
							Negative regulation of viral genome replication	GO:0045071
							Innate immune response	GO:0045087
	SLC25A6	ENSG00000169100	0.901	0.00107	0.0848	Solute carrier family 25 member 6	Active induction of host immune response by virus	GO:0046732
	EEF2	ENSG00000167658	0.902	0.00007	0.0341	Eukaryotic translation elongation factor 2	Hematopoietic progenitor cell differentiation	GO:0002244
	ST6GAL1	ENSG00000073849	0.932	0.00069	0.0735	ST6 beta-galactoside alpha-2,6-sialyltransferase 1	Humoral immune response	GO:0006959
Upregulated gene expression in CFS patients as compared to healthy controls	OGT	ENSG00000147162	1.087	0.00022	0.0461	O-linked *N*-acetylglucosamine (GlcNAc) transferase	Positive regulation of granulocyte differentiation	GO:0030854
	ATG7	ENSG00000197548	1.095	0.00026	0.0525	Autophagy related 7	Positive regulation of macroautophagy	GO:0016239
	LCP2	ENSG00000043462	1.095	0.00132	0.0915	Lymphocyte cytosolic protein 2	Immune response	GO:0006955
	PTPRE	ENSG00000132334	1.096	0.00057	0.0699	Protein tyrosine phosphatase, receptor type E	Transmembrane receptor protein tyrosine phosphatase signaling pathway	GO:0007185
	PRKCD	ENSG00000163932	1.102	0.00073	0.0735	Protein kinase C delta	Activation of phospholipase C activity	GO:0007202
	TNFRSF25	ENSG00000215788	1.108	0.00113	0.0872	Tumor necrosis factor receptor superfamily member 25	Inflammatory response	GO:0006954
	TLR8	ENSG00000101916	1.109	0.00132	0.0915	Toll like receptor 8	Toll-like receptor signaling pathway	GO:0002224
	BTN3A3	ENSG00000111801	1.124	0.00015	0.0406	Butyrophilin subfamily 3 member A3	T cell mediated immunity	GO:0002456
	S100A8	ENSG00000143546	1.142	0.00110	0.0859	S100 calcium binding protein A8	Cytokine production	GO:0001816
	NBEAL2	ENSG00000160796	1.143	0.00086	0.0752	Neurobeachin like 2	Hematopoietic progenitor cell differentiation	GO.0002244
	IFI16	ENSG00000163565	1.146	0.00066	0.0735	Interferon gamma inducible protein 16	Negative regulation of innate immune response	GO:0045824
							Adaptive immune response	GO:0002250
							Positive regulation of germinal center formation	GO:0002636
							Positive regulation of B cell proliferation	GO:0030890
							T cell costimulation	GO:0031295
							B cell costimulation	GO:0031296
							Positive regulation of T cell proliferation	GO:0042102
							Positive regulation of interferon-gamma biosynthetic process	GO:0045078
	BST1	ENSG00000109743	1.148	0.00070	0.0735	Bone marrow stromal cell antigen 1	Humoral immune response	GO:0006959
	JAML	ENSG00000160593	1.151	0.00000	0.0063	Junction adhesion molecule like	Neutrophil chemotaxis	GO:0030593
	TRIM25	ENSG00000121060	1.155	0.00035	0.0590	Tripartite motif containing 25	Viral process	GO:0016032
	FAM111A	ENSG00000166801	1.157	0.00008	0.0341	Family with sequence similarity 111 member A	Negative regulation of viral genome replication	GO:0045071
							Defense response to virus	GO:0051607
	CASP1	ENSG00000137752	1.165	0.00096	0.0799	Caspase 1	Regulation of inflammatory response	GO:0050727
	RIPK3	ENSG00000129465	1.167	0.00001	0.0136	Receptor interacting serine/threonine kinase 3	Regulation of T cell mediated cytotoxicity	GO:0001914
	CLEC2B	ENSG00000110852	1.179	0.00007	0.0336	C-type lectin domain family 2 member B	Regulation of immune response	GO:0050776
	IL1RN	ENSG00000136689	1.191	0.00016	0.0406	Interleukin 1 receptor antagonist	Negative regulation of cytokine-mediated signaling pathway	GO:0001960
	IRF9	ENSG00000213928	1.200	0.00041	0.0615	Interferon regulatory factor 9	Cell surface receptor signaling pathway	GO:0007166
	ADCY4	ENSG00000129467	1.206	0.00013	0.0395	Adenylate cyclase 4	cAMP-mediated signaling	GO:0019933
	PLSCR1	ENSG00000188313	1.209	0.00050	0.0677	Phospholipid scramblase 1	Platelet activation	GO:0030168
	PDE1B	ENSG00000123360	1.220	0.00011	0.0381	Phosphodiesterase 1B	Monocyte differentiation	GO:0030224
	APOBEC3A	ENSG00000128383	1.216	0.00004	0.0276	Apolipoprotein B mRNA editing enzyme catalytic subunit 3A	Defense response to virus	GO:0051607

A list of *all* 176 differentially expressed genes in the present study is given in Additional file 3: Table S3

**Fig. 3:**
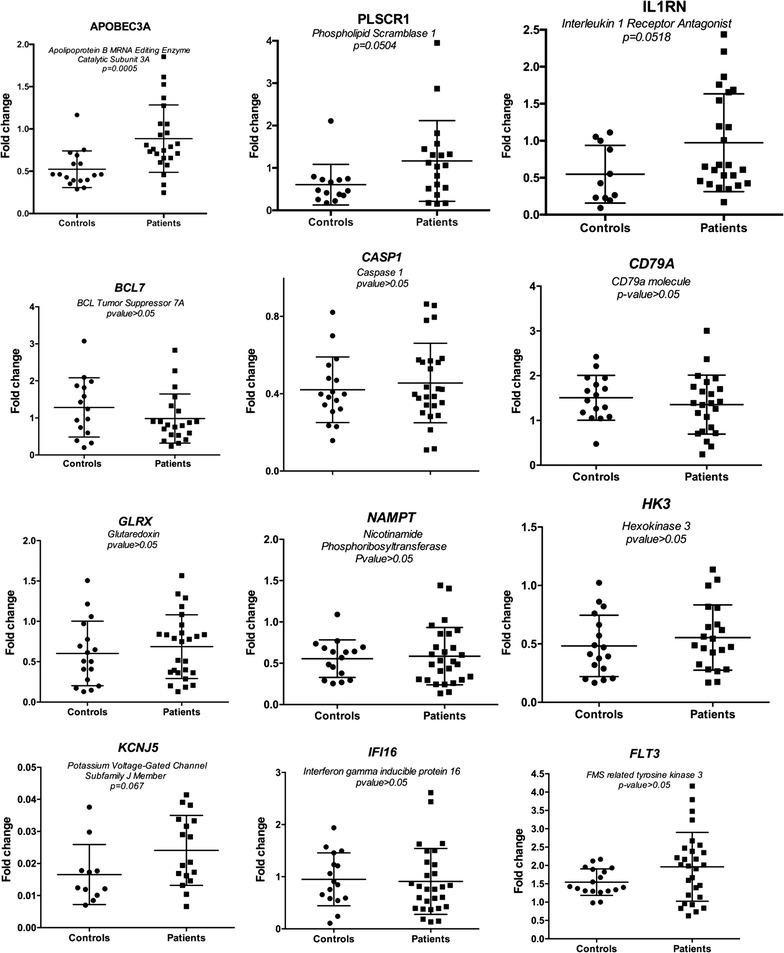
RT-qPCR results of 12 selected transcripts. CFS patients and controls are plotted on the *x axis* and relative fold change difference normalized against *GAPDH* is plotted on the *y axis*. For three transcript, the differential expression between patients and controls were below or close to the level of significance (*APOBEC3A*, p = 0.0005; *PLSCR1,* p = 0.0498; *IL1RN,* p = 0.0507)

A total of 12 genes were selected for further examination featuring RT-qPCR (Fig. 3). Because of the exploratory nature of this study, we wanted these selected genes to be as representative as possible for the RNA-Seq results as a whole: Three genes are related to B cells differentiation/survival (*CD79A*, *FTL3*) and B cell malignancies (*BCL7A*); in addition, these three genes are among the most under-expressed in the CFS group. Two genes are related to IL1 and IL17 signaling pathways (*IL1RN* and *GLRX1*, respectively). Two genes are annotated to inflammatory responses (*NAMPT*, *CASP1*). Three genes are related to innate antiviral defense (*APOBEC3A*, *IFI16*, *PLSCR1*). The final two genes (*HK3*, *KCJN5*) are the two most over-expressed in the CFS group. Ten of the transcripts were found to be differentially expressed in the same direction as in the RNA seq experiments; for three of the transcripts (*APOBEC3A*, *PLSCR1, IL1RN)*, the fold change differences were statistically significant or close to the level of significance (p = 0.0005, p = 0.0489, p = 0.0507, respectively, Mann–Whitney test). The fold changes measured between CFS patients and healthy controls were moderate, which is in accordance with the RNA-Seq data.

**Fig. 4:**
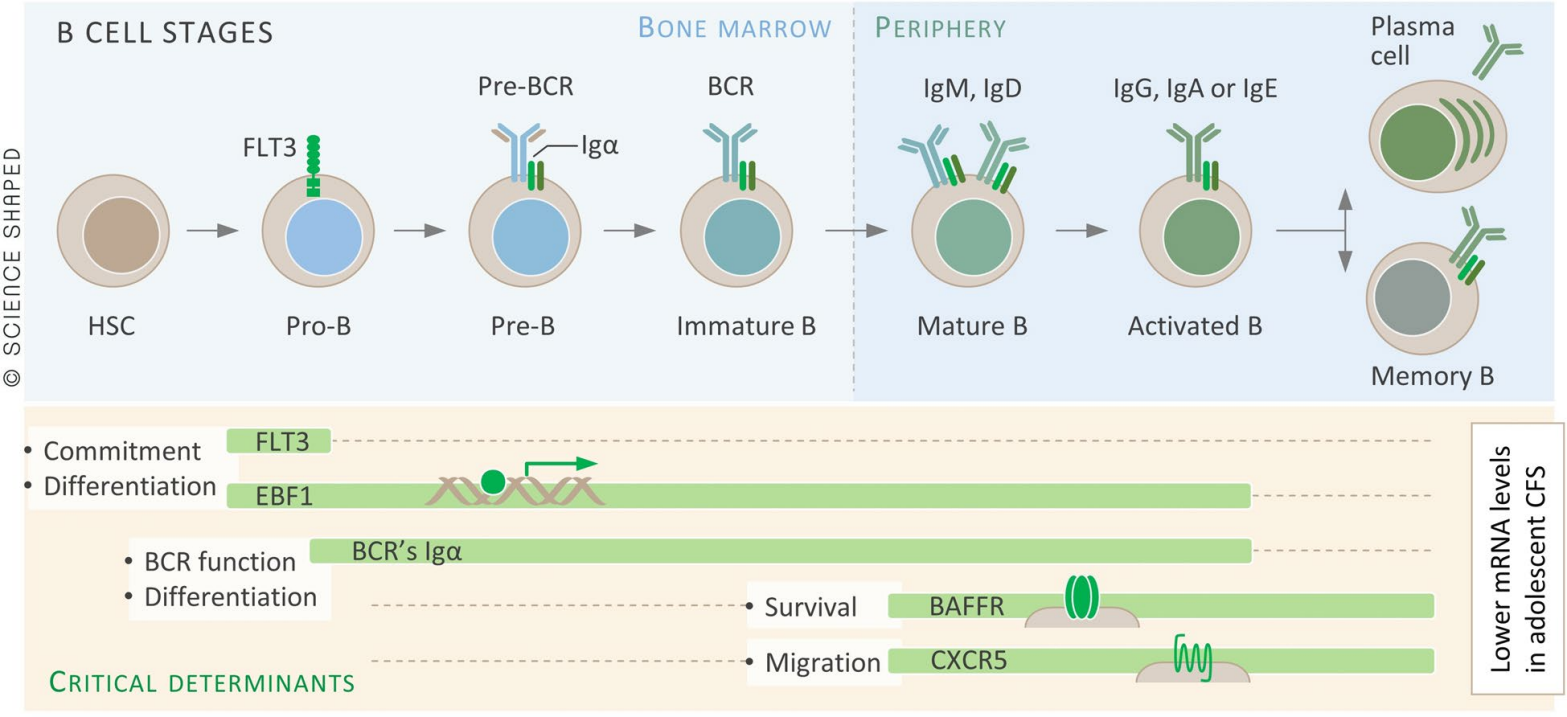
The RNA-Seq identified five down-regulated genes encoding proteins associated with B cell differentiation and survival. *FLT3* encodes FLT3 (fms-related tyrosine kinase 3), which is important during the very early stages of differentiation in the bone marrow of the hematopoietic stem cell into the Pro-B cell. *EBF1* encodes EBF (early B-cell factor 1), which is important during all stages of B cell differentiation except for the plasma cell. *CD79A* encodes Igα (immunoglobulin-associated alpha), which is a co-molecule in the membrane bound Pre-BCR and the BCR, and ensures a functional receptor. *TNFRSF13C* encodes BAFFR (B-cell activating factor receptor), which is important for the peripheral B cells to receive survival signal. *CXCR5* encodes CXCR5 [chemokine (C-X-C motif) receptor 5], which ensures that matured B cells migrate to B cell follicles of the spleen and Peyer patches. Assuming that the down-regulation of these genes is reflected at the protein and pathway level, our data suggest that the efficiency of B cell differentiation is impaired and that their survival is reduced in the CFS. *HSC* hematopoietic stem cell, *BCR* B cell receptor, *B* B cell, *Ig* immunoglobulin

Gene set enrichment analyses performed using Gene Ontology annotation by HumanMine and independent filtering, suggested that a large fraction of the DEGs (34 out of 176) were related to the immune system (Table 2). Five of the genes that were most down-regulated in the CFS group are associated with B cell differentiation and survival (Fig. 4, cf. above): *FLT3* (encoding FLT3, a tyrosine kinase), *EBF1* (encoding EBF, 1 early B cell factor 1), *CD79A* (encoding Igα, a co-molecule of the membrane bound B cell receptor (BCR) complex), *CXCR5* (encoding CXCR5, a chemokine receptor), and *TNFRSF13C* (encoding BAFFR, a receptor for B cell activating factor). Conversely, many of the genes that we found to be upregulated in CFS have a role in innate immunity and inflammation. Prominent examples include *CASP1* (encoding caspase 1), *CLEC2B* (encoding activation-induced C-type lectin), *PLSCR1* (encoding phospholipid scramblase 1), *IFI16* (encoding gamma-interferon-inducible protein 16), *PDE1B* (encoding cyclic nucleotide phosphodiesterase), *IRF9* (encoding interferon regulatory factor 9), *TLR8* (encoding toll-like receptor 8), and *APOBEC3A* (encoding a DNA editing enzyme).

### Downstream data analysis

Functional enrichment by ClueGO and visualization by Cytoscape identified a network of genes related to viral genome replication in the CFS group. Also, a downstream biological analysis using Ingenuity Pathway Analyses (IPA) confirmed that genes that are important for B cell differentiation and survival were down-regulated in the CSF patients. A search in IPA for mechanistic network enrichment of the upstream transcriptional regulators identified three top genes (Additional file 4: Table S4). The top upstream regulator identified was *IRF7*, which has functional couplings with STAT3 or STAT6 through TNF and IFN respectively [60]. The others were transcription factors: *SPI1* encodes a protein involved in myeloid and B cell lymphoid development, whereas *STAT6* encodes STAT6, which is activated by IL-4 and IL-13 and is important in signal transduction in many immune cells.

### Immunoglobulin classes and subclasses in CFS patients and healthy controls

As the DEGs suggested possible effects on B cell differentiation and survival among CFS patients, immunoglobulin classes and the IgG subclasses were analyzed across the two groups. Measurements of all immunoglobulin isotype fell within the linear range of the standard curve, except for one control sample in which IgG_3_ concentration was higher than the upper limit of detection. There were no across group differences among the serum levels of IgG_1_, IgG_2_, IgG_3_, IgG_4_, IgA, IgE, and IgM. Further characterization of B cell function in CFS could not be pursued, as viable PBMC that could be used for stimulation experiments were unavailable.

### Co-expression of genes and associations with immune, neuroendocrine and clinical markers within the CFS group

The principal component analyses (PCA) of all DEGs in the CFS group revealed that a 4-factor structure would account for 70% of the total variation. Inspection of the factor loadings revealed that several of the immune process annotated genes that were most differentially expressed across groups (including genes related to B cell differentiation and survival, and innate immunity) loaded on one factor (Additional file 5: Table S5), suggesting a possible co-expression pattern. Therefore, this factor, labelled “Factor 3” in the following, was selected for further explorative analyses.

In bivariate correlation analyses, factor 3 correlated positively with serum CRP-levels, granulocyte and monocyte count, plasma cortisol levels and indices of sympathetic nervous activity. There was a negative correlation with eosinophil count and indices of parasympathetic nervous activity. Finally, there was a slight association to symptoms of post-exertional malaise (p = 0.05), but not to any other clinical markers, including symptoms of depression and anxiety as well as physical activity (steps per day).

**Fig. 5:**
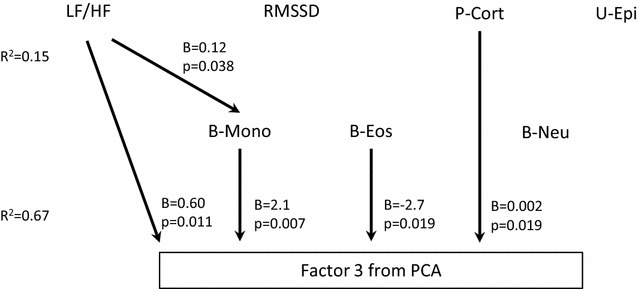
Multiple regression model on the associations between neuroendocrine markers (*upper row*), immune markers (*middle row*) and co-expression of genes as captured in Factor 3 from a principal component analysis (*lower row*). LF/HF, B-Mono, B-Eos and P-Cort are all independently and significantly associated with Factor 3, explaining 67% of the total variance. For LF/HF, B-Mono and P-Cort, the association is positive; for B-Eos the association is negative. In addition, LF/HF is significantly associated with B-Mono. *P* plasma, *U* urine, *B* blood, *LF/HF* low-frequency/high-frequency power of heart rate (an index of sympathetic vs parasympathetic balance), *RMSSD* square root of the mean squared differences of subsequent RR-intervals (an index of parasympathetic activity), *Cort* cortisol, *Epi* epinephrine, *Mono* monocytes, *Eos* eosinophils, *Neu* neutrophils, *PCA* principal component analysis, *B* regression coefficient (unstandardized), *R* ^*2*^ explained variance of the dependent variable in the multiple regression model

Based on results from bivariate correlation analyses as well as theoretical considerations, a multiple regression model was explored. The final model explained 67% of Factor 3 total variance (Fig. 5). LF/HF ratio (an index of sympathetic vs parasympathetic balance), blood monocyte count, and plasma cortisol levels were positively associated with Factor 3, whereas blood eosinophil count was negatively associated with Factor 3. Furthermore, LF/HF ratio was positively associated with blood monocyte count.

### Associations of individual transcripts with immune, neuroendocrine and clinical markers within the CFS group

To further explore associations between gene expression and immune, neuroendocrine and clinical markers, transcripts that loaded on Factor 3 and in addition were annotated to immune processes (cf. Table 2) were selected. Three of the selected genes (*CD79A, TNFRSF13C, CXCR5)* are related to B cell differentiation and survival; they loaded negatively on Factor 3 (Additional file 5: Table S5) and were also less expressed in the CFS group. Three other genes (*CASP1, PLSCR1, IFI16*) are related to regulation of innate immune responses; they loaded positively on Factor 3 and were also overexpressed in the CFS group.

**Fig. 6:**
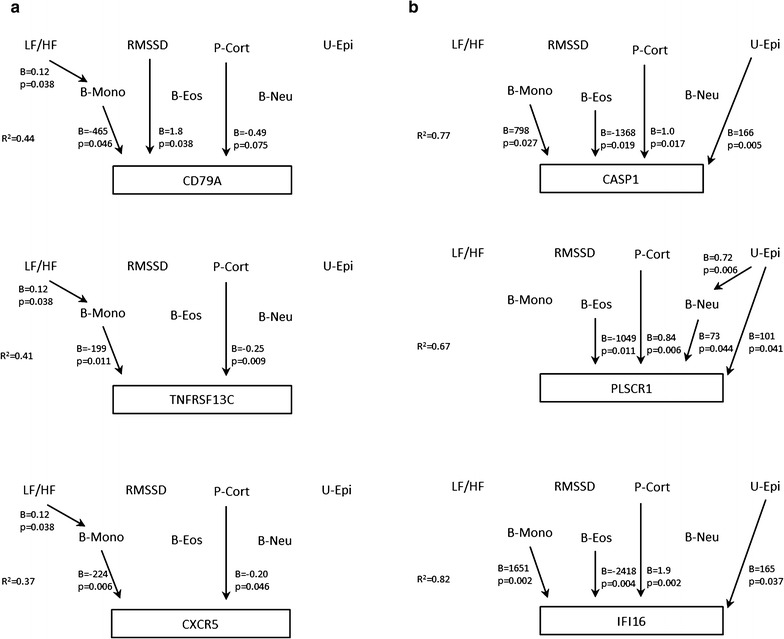
Multiple regression models on the associations between neuroendocrine markers, immune markers and single gene transcripts within the CFS group. **a** Three genes related to B cell differentiation and survival, with negative loadings of Factor 3 and down-regulated expression in the CFS-group as compared to healthy controls. **b** Three genes related to innate immunity, with positive loadings of Factor 3 and up-regulated expression in the CFS group as compared to healthy controls. *P* plasma, *U* urine, *B* blood, *LF/HF* low-frequency/high-frequency power of heart rate (an index of sympathetic vs parasympathetic balance), *RMSSD* square root of the mean squared differences of subsequent RR-intervals (an index of parasympathetic activity), *Cort* cortisol, *Epi* epinephrine, *Mono* monocytes, *Eos* eosinophils, *Neu* neutrophils, *PCA* principal component analysis, *B* regression coefficient (unstandardized), *R* ^*2*^ explained variance of the dependent variable in the multiple regression model

The transcript of all the three genes related to B cell differentiation and survival tended to correlate negatively with blood neutrophil count, blood monocyte count, serum CRP, plasma cortisol, LF/HF ratio and symptoms of post-exertional malaise, and positively with blood eosinophil count and RMSSD (Additional file 6: Table S6). An opposite pattern was observed for the three genes related to innate immunity; in addition they were positively associated with urine epinephrine, but not with clinical symptoms. In multiple regression models, a homogeneous picture was observed regarding the three B cell related transcripts (Fig. 6a): there was a significant negative association to plasma cortisol levels and a significant positive association to blood monocyte count, which in turn was positively associated with LF/HF ratio. For the transcripts related to innate immunity, the picture was more heterogeneous (Fig. 6b), but all were negatively associated with eosinophil count and positively associated with plasma cortisol and urine epinephrine levels.

## Discussion

The main findings of this study are: (a) A total of 176 genes are differentially expressed in whole blood across adolescent CFS patients and healthy controls after adjusting for age and gender differences (FDR 10%); in CFS, there is down-regulation of genes related to B cell differentiation and survival, and upregulation of genes related innate antiviral responses and inflammation. (b) Within the CFS group, the differentially expressed genes are associated with neuroendocrine markers of altered HPA-axis and autonomic nervous activity, as well as with symptoms of post-exertional malaise.

The down-regulated genes related to B cell differentiation and survival included the genes mentioned above: *EBF1, CD79A*, *CXCR5*, *TNFRSF13C*, and *FLT3*. The FLT3 protein acts as a cell-surface receptor and is a regulator for the differentiation, proliferation and survival of B cell progenitor cells in the bone marrow [61]. The EBF1 protein is a transcription factor that is expressed in B cells at all stages of their differentiation except for fully differentiated plasma cells [62]. The Igα encoded by *CD79A* is a co-molecule of the BCR complex and ensures that the signal cascade for recognition of antigen is sent. This is necessary for internalization of the BCR-antigen complex and further processing and presentation of antigen peptides on the B cell surface [63]. The chemokine receptor CXCR5 is important for migration of B cells into secondary lymphoid organs [64]. The B cell activating factor receptor (BAFFR) encoded by *TNFRSF13C* enhances mature B cell survival and controls peripheral B cell population [65]. Taken together, our data suggest that the efficiency of B cell differentiation is impaired and that their survival is reduced in the CFS patients (Fig. 4).

As for upregulated innate immunity genes, a number was related to viral defence mechanisms. *APOBEC3A* was enriched in the negative regulation of viral genome replication together with *PLSCR1* and *FAM111A* (a chromatin-associated DNA clamp required for proliferating cell nuclear antigen loading on replication sites). The enzyme encoded by *APOBEC3A* deaminates foreign DNA as part of viral clearance [66], whereas phospholipid scramblase 1 (encoded by *PLSCR1*) was observed to play a role in enhancement of IFN response and increase expression of antiviral genes in mice [67]. This network was in turn connected to IFI16, Gamma-interferon-inducible protein 16, which is a sensor for intracellular DNA and a mediator of IFN induction. Other genes that were found to be related to IFN signaling were the genes encoding interferon regulatory factor 9 (*IRF9*) and *TLR8*. The Interferon regulatory factor 9 is a component of the interferons stimulated gene factor 3 complex that is involved in positive regulation of type I interferon gene [68]. TLR8 is an endosomal receptor which acts against foreign ssRNAs by intracellular signalling through NF-κB or IRF7 pathways [69].

Other upregulated innate immunity genes were related to inflammation: Caspase 1 (encoded by *CASP1*), having a central role in the formation of inflammasomes and other inflammatory-related responses [70]; activation-induced C-type lectin (encoded by *CLEC2B*), which promote the cross-talk between monocytes and NK-cells [71]; and cyclic nucleotide phosphodiesterase encoded by *PDE1B*, which is important for the cellular response to granulocyte macrophage colony-stimulating factor [72].

Down-regulation of genes important for B cell differentiation and survival in CFS, as suggested by the present study, comply with a previous CFS studies: Recently, increased levels of the B lymphocyte activating factor of the tumor necrosis family (BAFF) was reported in adults with CFS [73]. We speculate that this might be a compensatory mechanism as BAFF is a ligand for BAFFR encoded by *TNFRSF13C*, which is one of the most suppressed genes among CFS patients in the present study. Taken together, these results might indicate a role for B cells in CFS pathophysiology, as is supported from studies of cellular immunology: Brenu and co-workers reported a decrease in immature B cells and an increase in memory B cells among CFS patients [74], whereas Bradley and co-workers [75] and Mensah and co-workers [76] found subtle distortions in the proportion of B cell subsets. Alterations of immunoglobulin levels in CFS have also been reported [77], but was not identified in the present material, which is not surprising given the strong propensity of compensatory mechanisms to ensure normal immunoglobulin levels in circulation despite changes in B cell function [78].

Up-regulation of genes related to innate antiviral responses has, to our knowledge, not been consistently reported previously in CFS, not even in cohorts suffering from chronic fatigue following long-lasting viral infections [25]. Our data point to functionally connected genes and pathways involved in innate immunity responses as differentially expressed in the CFS group and might suggest less efficient viral clearance or reactivation of latent viruses such as members of the herpes virus family, in the CFS group [79]. Of note, the herpes virus Epstein-Barr virus (EBV) is a well-known trigger of CFS in adolescents [80]. The possible presence of inefficient viral clearance or virus reactivation, and whether intracellular signaling cascades activated by long-lasting viral infections may be a contributor to CFS pathophysiology, warrant further studies. A model from Thorley-Lawson suggested that EBV uses a pathway similar of B cell survival and B cell differentiation in order to establish its infection, persistence and replication [81]. Loebel and co-workers assumed that a frequent EBV reactivation or impaired control of EBV was a result of the diminished EBV-specific memory B cell response in CFS patients [82]. Therefore we speculate that in some patients, CFS is characterised by persistent EBV-host interactions. Based on the observation of altered B cells differentiation and B cell survival signature, in future experiments, we aim to validate the finding by measuring B cell responsiveness to stimulator such as EBV virus antigens [viral capsid antigen (VCA) and EBV nuclear antigen 1 (EBNA-1)] alone or after exposure to the neuroendocrine hormones.

Up-regulation of genes related to inflammation in the CFS group, which is corroborated by the positive correlation between “Factor 3″ and serum CRP levels, comply with previous CFS studies reporting elevation of proinflammatory cytokines in adult CFS [12, 13, 83]. Interestingly, a recent study of gene expression in NK cells of CFS patients showed upregulation of *RIPK3* [84], in line with the present data (Additional file 3: Table S3); this gene encodes a kinase that plays a vital role in inflammasomes and IL-1β signaling. However, a previous analysis of cytokine levels in the present material did not relieve any differences in CFS patients as compared to healthy controls [15]. Thus, a skewing of the immune response towards inflammation appears to be subtle, or even indirect, complying with other studies of gene expression reporting small or moderate fold changes in inflammatory related gene transcripts [16, 25].

The strong association between “Factor 3” with neuroendocrine markers within the CFS group is a novel finding. Although causal interferences cannot be made from our cross-sectional design, the results are in line with the “sustained arousal” model of CFS which suggests that immune alterations are secondary to neuroendocrine alterations [37]. This potential mechanism complies with findings in studies of neuro-immunomodulation: Sympathetic nervous activity has complex effects on B cells, monocytes and several other immune cells through adrenergic receptors that in turn promote alteration of gene expression [35]. Parasympathetic nervous activity has a well-described anti-inflammatory effect based upon gene expression alterations of spleen macrophages [34]. The glucocorticoid effects on immunity are extensive [33], and might in addition be abnormal in CFS, as some studies have indicated a fundamental alteration of glucocorticoid signaling [32, 85].

Taken together, the results of the present study might indicate a skewing of the immune responses from adaptive to innate immunity promoted by the combined effect of HPA axis alteration and sympathetic vs. parasympathetic predominance in CFS patients. We speculate that “Factor 3” in the present data set encapsulates this skewing, being negatively associated with transcripts regulating B cell differentiation and survival, and positively associated with transcripts involved in inflammation and innate antiviral defense. Interestingly, such a skewing shares some similarities with the concept of “Conserved Transcriptional Response to Adversities” (CTRA) [86]. Recent evidence suggests that CTRA is promoted by increased sympathetic nervous activity to the bone marrow, altering myeloid cell numbers and function and promoting functional glucocorticoid desensitization [87]. This complies with the present findings of autonomic nervous activity indices, blood monocyte and eosinophil counts, as well as plasma cortisol level being strongly associated with “Factor 3” (Figs. 5, 6).

The present study did not demonstrate strong associations between gene expression profiles and clinical markers; this lack of association to clinical symptoms is in line with other studies [24, 25]. Specifically, there was no correlation between gene transcripts and symptoms of inflammation within the CFS group, confirming previous findings [15]. However, the present data did suggest an association between differential gene expression and symptoms of post-exertional malaise, which is considered a hallmark of the CFS phenotype [1]. This association was primarily evident for the transcripts related to B cell differentiation and survival (Additional file 6: Table S6), an observation that warrants further studies. The lack of association between “Factor 3” and depressive symptoms, trait anxiety and steps per day suggests that the findings are not confounded by the co-existence of emotional problems nor physical inactivity in the CFS group.

### Study strengths and limitations

A strength of this study is the HTS based methods combined with extensive clinical phenotyping. The background data show that the subsets of participants in the present study are comparable to the entire NorCAPITAL cohort (Additional file 2: Table S2). However, the numbers of subjects are relatively low, and the wide inclusion criteria might have obscured results pertaining to a subgroup; unfortunately, the study did not have sufficient statistical power to allow meaningful subgroup analyses. In addition, the relatively strict p value cut off of ≤0.1 (after multiple-testing adjustment) for identifying DEGs might increase the risk of type 2-errors. However, previous studies of the NorCAPITAL data set do not suggest subgroup differences [15, 32, 39, 53]. Furthermore, important background factors such as BMI, smoking status and alcohol consumption do not differ across patients and controls, reducing the risk of confounding effects [39]. There was a relatively poor correspondence between RNA seq results and RT-qPCR results; reasons for this discrepancy might be different primers and different normalization methods between RNA seq and RT-qPCR, as well as low concentration of remaining cDNA after RNA seq. Also, the study might have benefitted from a more stringent approach for selecting genes for RT-qPCR analyses. In addition, the design of the NorCAPITAL project did not allow analyses of correlation between mRNA levels and protein levels. Another limitation is that we did not assess gene expression responses to exercise or other stimuli (such as fatigue provoking mental activity), which might have provided important additional information [19]. Furthermore, the RNA seq analysis was not corrected for the different cell populations in whole blood. The investigational program in the NorCAPITAL project did neither include subtyping nor biobanking of peripheral blood cells; thus, validation of the gene expression findings with flow cytometer analyses or functional assays was not possible in the present study. Future studies should include deep phenotyping of the peripheral cell populations and analysis of their effector functions. Further studies should also be powered to allow subgroup analyses, as well as ensure robust validation of the findings.

## Conclusion

Adolescent CFS is characterized by differential gene expression pattern in whole blood suggestive of impaired B cell differentiation and survival and enhanced innate antiviral responses and inflammation. This expression pattern is associated with neuroendocrine markers of altered HPA axis and autonomic nervous activity, and with symptoms of post-exertional malaise. Taken together, the results contribute to the understanding of CFS disease mechanism, which in turn is a prerequisite for development of improved diagnostic procedures and therapeutic interventions. Also, the results are in in line with the “sustained arousal”-model of CFS disease mechanisms, in which a causal relationship between neuroendocrine changes and immune alterations is suggested [37]. This possible causality, as well as the association to CFS clinical symptoms and the specific role of altered B cell function, should be explored in further studies.

## Additional files

**Additional file 1: Table S1.** Primer names and sequences for the RT-qPCR experiments.

**Additional file 2: Table S2.** Background characteristics of the chronic fatigue syndrome (CFS) group and the healthy control (HC) group in the present study compared with the characteristics of all CFS patients and HC in the NorCAPITAL dataset.

**Additional file 3: Table S3.** Differentially expressed genes and their annotated proteins or gene products in CFS patients as compared to healthy controls, adjusted for age and gender differences across groups and sorted according to foldchange.

**Additional file 4: Table S4.** Upstream transcriptional regulators for the observed dataset based on ingenuity pathway analyses (IPA) mechanistic network enrichment.

**Additional file 5: Table S5.** Principal component analysis (PCA) with varimax rotation in the CFS group.

**Additional file 6: Table S6.** Pearson correlation between single gene transcriptional counts and selected immune, neuroendocrine and clinical markers within the CFS group. Genes are sorted according to differential expression foldchange (column 2) as compared with healthy controls.

## Abbreviations

CFS: chronic fatigue syndrome
NorCAPITAL: The Norwegian Study of Chronic Fatigue Syndrome in Adolescents: Pathophysiology and Intervention Trial
HPA axis: hypothalamus–pituitary–adrenal axis
IL: interleukin
TFN: tumor necrosis factor
PBMC: peripheral blood mononuclear cells
CRP: C-reactive protein
HTS: high throughput sequencing
RNA-Seq: RNA sequencing
QC: quality control
DEG: differentially expressed genes
RT-qPCR: real-time quantitative polymerase chain reaction
IPA: ingenuity pathway analysis
PCA: principal component analysis
HPLC: high-performance liquid chromatography
HRV: heart rate variability
LF: low frequency
HF: high frequency
RMSSD: square root of the mean square difference of successive RR-intervals
CFQ: Chalder Fatigue Questionnaire
MFQ: Moods and Feelings Questionnaire
BCR: B cell receptor
BAFFR: B cell activating factor receptor
CTRA: conserved transcriptional response to adversities
